# Combining THz laser excitation with resonant soft X-ray scattering at the Linac Coherent Light Source

**DOI:** 10.1107/S1600577515005998

**Published:** 2015-04-11

**Authors:** Joshua J. Turner, Georgi L. Dakovski, Matthias C. Hoffmann, Harold Y. Hwang, Alex Zarem, William F. Schlotter, Stefan Moeller, Michael P. Minitti, Urs Staub, Steven Johnson, Ankush Mitra, Michele Swiggers, Peter Noonan, G. Ivan Curiel, Michael Holmes

**Affiliations:** a Linac Coherent Light Source, SLAC National Accelerator Laboratory, 2575 Sand Hill Road, Menlo Park, CA 94025, USA; b Department of Chemistry, Massachusetts Institute of Technology, Cambridge, MA 02139, USA; c Swiss Light Source, Paul Scherrer Institut, 5232 Villigen, Switzerland; d ETH Zurich, Institute for Quantum Electronics, Wolfgang-Pauli-Strasse 16, 8093 Zurich, Switzerland

**Keywords:** FEL, X-ray diffraction, THz, instrumentation

## Abstract

This paper describes the development of new instrumentation at the Linac Coherent Light Source for conducting THz excitation experiments in an ultra high vacuum environment probed by soft X-ray diffraction. This consists of a cantilevered, fully motorized mirror system which can provide 600 kV cm^−1^ electric field strengths across the sample and an X-ray detector that can span the full Ewald sphere with in-vacuum motion. The scientific applications motivated by this development, the details of the instrument, and spectra demonstrating the field strengths achieved using this newly developed system are discussed.

## Abstract 2

This paper describes new instrumentation developments at the LCLS for materials studies using THz laser excitation and resonant soft X-ray scattering.

## 1. Introduction   

The recent development of the X-ray free-electron laser (FEL) has opened up numerous new scientific opportunities, paving the way for many scientific breakthroughs in its short history. For example, FEL experiments have offered new insights in structural biology enabling a better understanding of African sleeping sickness (Redecke *et al.*, 2013 ▶) and of the proteins responsible for photosynthesis at room temperature (Kern *et al.*, 2013 ▶). The soft X-ray range affords further unique opportunities in an array of scientific fields, including astrophysics (Bernitt *et al.*, 2012 ▶), planetary science (Vinko *et al.*, 2012 ▶) and surface chemistry (Dell’Angela *et al.*, 2013 ▶). One area, however, which has shown increased potential is in the area of understanding materials. In particular, resonant soft X-ray diffraction has proven to be a valuable tool to look at long-range ordering of different order parameters. An adaptation of this tool has allowed researchers to gain time-resolved information of relevant order parameters in condensed matter systems in response to dynamic stimuli in materials such as CuO (Johnson *et al.*, 2012 ▶), manganites (Tobey *et al.*, 2012 ▶) and nickelates (Caviglia *et al.*, 2013 ▶; Chuang *et al.*, 2013 ▶). Recent work has even utilized pulsed pumping schemes at specialized photon energies to exclusively excite phonons (Först *et al.*, 2011 ▶, 2013 ▶) as a route to material control. However, using low-photon-energy excitation to study the intricate workings of quantum materials (Kubacka *et al.*, 2014 ▶) is an area which has so far been under-utilized, but remains quite promising.

Radiation in the THz regime (∼0.4–40 meV) (Kampfrath *et al.*, 2013 ▶; Hwang *et al.*, 2014 ▶) lies in a unique photon energy range for potential investigations in deciphering the nature of competing interactions in a material at nearly degenerate energies. The low photon energies are associated with many low-energy excitations in solids (Basov *et al.*, 2011 ▶). The combination of strong THz excitation sources and the advent of fourth-generation light sources has enabled the study of long-range order at atomic length scales and ultrafast timescales (Hoffmann & Turner, 2012 ▶) and is one of the motivations for the developments discussed in this paper.

One challenge with measuring soft X-ray diffraction in an ultra high vacuum environment is the incorporation of an in-vacuum diffractometer. Doing so enlarges the vacuum chamber, especially when the chamber volume is designed to span the full range of the Ewald sphere with a large area detector. Furthermore, a large optical element very close to the sample is needed to strongly focus the low-photon-energy pump pulses near the diffraction limit, thus maximizing the intensity at the sample. Typically a small-diameter vacuum chamber is used with the optical element outside the chamber. THz pump/soft X-ray probe experiments have been quite challenging because of the competing needs of a large vacuum chamber volume for the diffractometer and small chamber volume for strong focusing of the excitation pulse.

We have solved this problem by designing a focusing system inside a large-diameter vacuum chamber. By cantilevering a 2 inch-diameter optic from the side of the vacuum chamber, we have allowed for full control of six degrees of freedom for the focusing system alignment. As a result, we can now provide THz pumping at electric field strengths of up to 600 kV cm^−1^ (corresponding to magnetic field strengths of up to 200 mT) on a sample for resonant soft X-ray scattering experiments. The chamber has also been designed to prevent interference from the movement of an avalanche photodiode (APD) detector assembly with the optics. The assembly can move over a hemisphere about the sample to measure all possible diffraction angles at a distance of 14 cm 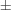 3.8 cm. This new design allows for THz pump/soft X-ray diffraction probe experiments with probing in any Bragg geometry. The angular range of detection is limited only by the size of the shadow of the focusing mirror placed in front of the sample onto the detector plane.

## 2. Resonant scattering at FELs   

The promise offered by using resonant scattering at soft X-ray energies for FEL research is astounding. X-ray scattering has been used to understand distinct types of long-range ordering by using resonant enhancement. This is especially useful in the soft X-ray range because many of the *L*-edges for important materials, such as transition metal oxides, lie in the soft X-ray range. The *L*-edges in these types of materials are important because they give access to types of order that involve the *d*-electrons, those relevant for emergent behavior arising from interactions of the orbital, charge or magnetic degrees of freedom. Furthermore, what makes this technique interesting for FEL research is the large degree of coherence (Vartanyants *et al.*, 2011 ▶), which means coherent scattering methods could also be used to study dynamics for these types of materials, such as in magnetic or orbital domain fluctuations (Konings *et al.*, 2011 ▶; Seu *et al.*, 2010 ▶; Turner *et al.*, 2008 ▶).

Central to FEL experiments on materials is the pump–probe technique, where ordering is enabled/disrupted by a pump pulse, and then the change of that order is monitored by a probe pulse – in this case an ultrafast soft X-ray pulse. This enables the study of distinct charge, orbital and spin order following an excitation mechanism, allowing for a full study of long-range-ordering dynamics. Comparing different types of interaction strengths in non-equilibrium conditions is the real strength of this method because it allows one to unravel the different ordering mechanisms by separating them in the time domain, offering prospects for their control. Though much excellent work has been done using non-selective optical pump methods (Staub *et al.*, 2014 ▶; de Jong *et al.*, 2013 ▶; Lee *et al.*, 2012 ▶), recent work has shown the importance of selective excitation to clarify the physics of the mechanisms being studied (Fausti *et al.*, 2011 ▶). For instance, the lattice of a superconductor can be resonantly pumped while observing the response of the charge density wave state (Först *et al.*, 2014*a* ▶), or of the charge order and of the crystal lattice structure itself (Först *et al.*, 2014*b* ▶). In the case of quantum materials many of the interactions compete on even lower energy scales, on the order of tens of meV. This motivates the use of lower-energy laser sources because resonant pumping can be performed to separate these mechanisms. By tuning to different types of modes, the physics in complex systems can be unraveled on ultrafast timescales such as in the recent study of electromagnons (Kubacka *et al.*, 2014 ▶).

## 3. Instrumentation   

The instrumental developments addressed in this paper were made to an existing diffraction chamber (Doering *et al.*, 2011 ▶) which is now permanently located at the Linac Coherent Light Source (LCLS). The system incorporates a 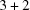-circle diffractometer which can move a detector anywhere along the surface of the Ewald sphere. Currently, APDs are being used to detect the soft X-ray photons; however, we plan on improving the setup by utilizing a multi-channel plate (MCP) as well.

The sample goniometer has three-axis linear motion, and all three angular degrees of freedom (θ, χ and ϕ) available for adjustment. In addition, the sample is attached to a liquid-helium-flow cryostat to adjust the temperature of the sample from 15 to 350 K. The whole diffraction chamber is mounted to a six-strut system for alignment and includes a load lock for interchanging samples without venting the system. The chamber can maintain pressures of 10
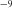
 Torr without cooling. The entire system has been integrated with an operating system to fully control all aspects of the system while taking measurements remotely from outside the enclosed X-ray hutch. The system is one of the endstations for use by the general user community at the SXR instrument (Schlotter *et al.*, 2012 ▶). This SXR instrument delivers pulses up to about 1 mJ per pulse to the endstation, depending on the photon energy range of 285 eV–2 keV (Tiedtke *et al.*, 2014 ▶). The degree of monochromaticity can be tuned (Heimann *et al.*, 2011 ▶) with pulse durations ranging from less than 10 fs up to a few hundred fs, and the instrument includes an array of timing diagnostics (Krupin *et al.*, 2012 ▶; Beye *et al.*, 2012 ▶), absolute pulse energy calibration (Moeller *et al.*, 2015 ▶), and a KB optical system to adjust the X-ray spot size down to a few microns (Chalupsky *et al.*, 2011 ▶). For more information on the capabilities of the SXR instrument and an overview of some of the experiments that have been carried out using it, *cf*. Dakovski *et al.* (2015 ▶).

**Figure 1:**
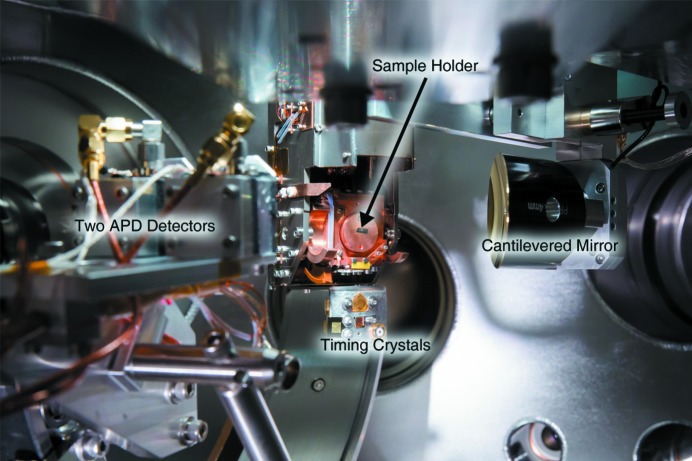
Photograph showing the new in-vacuum optics and motorization together with the cryostat and sample manipulator with the detector arm. This view is in the middle of the two incoming beams, 45° from both the incoming THz beam as well as to the incoming X-ray beam which travels through the small hole in the back of the cantilevered focusing mirror. The view is parallel to the support which cantilevers the setup from the wall of the vacuum chamber in the center of the interaction region. Also seen are the detectors on the support arm and the timing crystals which hang below the sample.

The newly developed instrument is shown in Figs. 1 ▶ and 2 ▶. The main focus of the current setup is the positioning and control of a large THz focusing mirror assembly very close to the sample in-vacuum. We address this by mounting a 2 inch parabolic mirror with a 2 inch effective focal length nominally 2.25 inches from the sample to optimize a slightly diverging beam at the center of the chamber. This directs a strong THz excitation pulse onto the sample for a pump–probe experimental geometry. Using a short-focal-length optic very close to the sample enables near-diffraction-limited THz spot sizes, as small as 300 µm for DSTMS crystal THz generation (see below), and has increased the available THz field strength by a factor of 20 compared with previous measurements where the focusing optics were located outside the vacuum chamber. The focal distance, as well as the two directions transverse to the beam, can all be optimized remotely by three orthogonal, in-vacuum linear stages (Micos VT-80, custom). The focusing optic has a small hole through the center, approximately 3 mm in diameter, to allow the X-ray beam to illuminate the sample in a collinear geometry with the THz excitation. The in-vacuum linear stages allow for alignment of the hole to the beam, as well as optimization of the focal distance from the optic to the sample. All three stages have a range of 100 mm (P/N 6230Z018), except for the vertical motion which has a range of 25 mm (Micos VT-80 custom, P/N 62305050), limited by the chamber height. The mirror system is cantilevered off the side of the chamber to place the mirror near the center of the chamber and the focus axis collinear to the incoming X-ray beam.

The instrument also has a control system to change the angles of the THz focusing mirror assembly remotely. The most important rotation is the degree of freedom that translates the THz focus vertically across the sample, because it must be set such that the collinear X-rays are unobstructed to the sample as well as ensuring spatial overlap between the THz and X-ray beams. This motion is controlled *via* a 2 inch-diameter in-vacuum rotary stage (Newport Agilis Piezo rotation stage: P/N AG-PR100V6). The degree of freedom that rotates the focus across the sample, horizontally, is controlled by a picomotor (NewFocus picomotor: P/N 8301-UHV). The adjustment to this motion is not as critical and is typically set at the start of the experiment and not moved as frequently. The third angle is the roll of the focusing mirror and is set manually.

There are a number of ways to generate high-field THz pulses (Hwang *et al.*, 2014 ▶), including using the LCLS itself (Daranciang *et al.*, 2011 ▶). We use a nonlinear organic crystal, 4-*N*,*N*-dimethylamino-4-*N*-methyl-stilbazolium 2,4,6-trimethylbenzenesulfonate (DSTMS), as a medium for nonlinear frequency conversion (Yang *et al.*, 2007 ▶). Strong THz pulses are achieved *via* optical rectification of mJ-level pulses centered around 1.5 µm in wavelength (Hauri *et al.*, 2011 ▶). A Ti:sapphire-based ultrafast laser system synchronized to the FEL delivers up to 15 mJ of 50 fs (FWHM) pulses centered at 800 nm to a high-power optical parametric amplifier (OPA) (Minitti *et al.*, 2015 ▶). The signal of the OPA was tuned to 1.5 µm at 50 fs with a maximum output of 2.0 mJ per pulse to enable efficient THz generation in a 400 µm-thick DSTMS crystal. This produces nearly single cycle THz pulses with energies of a few µJ and peak brightness at 2 THz.

The THz-generation crystal is situated in a nitrogen-purged environment outside the main vacuum chamber. The THz beam is transported into the chamber through a polymer window that is transparent for both THz and visible light (Topas Advanced Polymers, part 6013S-04: Cyclic Olefin Copolymer), simplifying the alignment procedure. The field strength is measured by electro-optical sampling using an additional 800 nm probe beam overlapped with both the X-rays and the THz beam at the sample position. The polarization optics and detection for the 800 nm probe beam are located on the downstream end of the chamber, outside the vacuum system as in Kubacka *et al.* (2014 ▶). For more information on the general laser capabilities at the LCLS, *cf*. Minitti *et al.* (2015 ▶).

With the current setup, we can produce a measured peak THz electric field of 0.6 MV cm^−1^ using the new optical in-vacuum setup shown in Fig. 3 ▶, comparable with what is generated on a traditional optical setup (Yeh *et al.*, 2007 ▶). This was using a gold-coated aluminium mirror with a numerical aperture of 1/6. Field-strength calibration was performed *via* electro-optical sampling by inserting a 100 µm-thick (110)-cut GaP crystal in-vacuum mounted on the cryostat. We are currently developing methods to further increase the field strength by using different materials for THz generation (Hoffmann & Fülöp, 2011 ▶).

**Figure 2:**
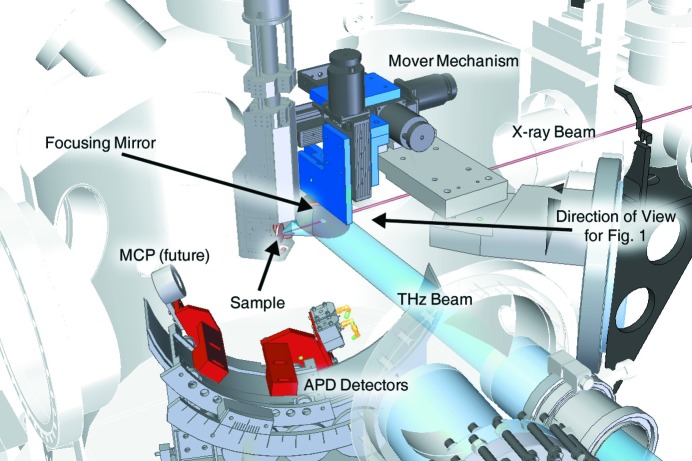
The model of the upgrade for the new THz excitation instrument. The blue components are the mechanisms designed to move the mirror in three dimensions and adjust the angle of the mirror with three angular degrees of freedom with respect to the incoming THz beam (shown in blue). The red components show the X-ray detectors, both the APDs and the MCP (future), used to detect scattered X-rays (shown in red).

**Figure 3:**
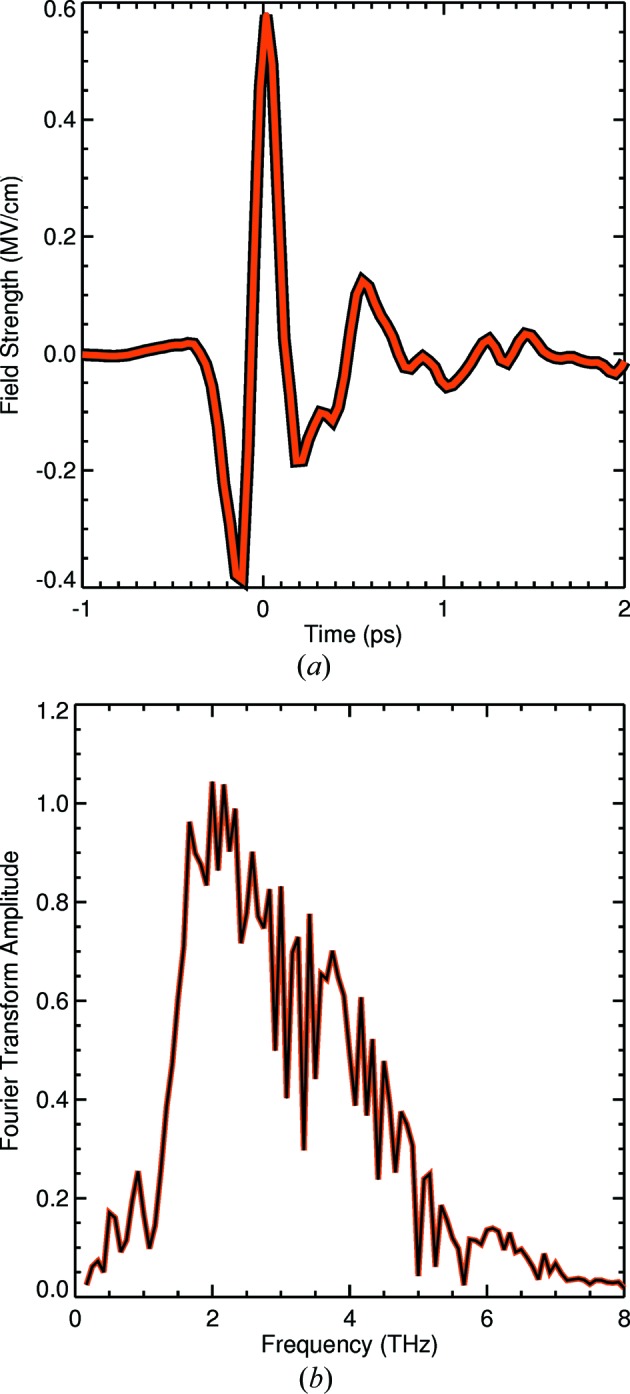
Temporal electric field profile (*a*) of the THz pump pulse, measured by electro-optic sampling in gallium phosphide. The peak electric field is 0.6 MV cm^−1^ corresponding to a peak magnetic field of 200 mT. (*b*) The amplitude spectrum of the THz pump pulse in (*a*), showing the frequency content of the pulse.

An additional challenge with such a setup can be to measure the diffracted X-ray beam intensity adequately at the requisite point in reciprocal space without interfering with the focusing optics. Currently, two APDs are used for detection and are built in a custom-designed encasement which prevents unwanted optical and radio frequency signals. With a newly designed detector arm, the full range of motion can be explored with two adjacent APD detectors which can incorporate a slit or filter to block optical radiation. The distance to the sample can be adjusted from 10.2 cm to 17.8 cm depending on the nature of the experiment. The new design (see Fig. 2 ▶) places the detector in the plane behind the focusing optic such that it can move freely behind the optical system with no interference. A fast area detector is being developed at the SLAC National Accelerator Laboratory (Blaj *et al.*, 2015 ▶), which will have 0.5 megapixels over 2 cm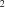, and a readout frame rate of 120 Hz with 20 electrons of readout noise. With this technology, we will be developing a specific detector to incorporate into this setup for two-dimensional measurements, such as time-resolved coherent diffraction measurements in materials. Additionally, we are planning to have an MCP mounted on the detector stage for low-photon-count experiments.

## 4. Conclusion   

New instrumentation has recently been commissioned at the SXR instrument to perform low-photon-energy THz pump/resonant soft X-ray diffraction probe measurements. With fully motorized in-vacuum optics, THz electric field strengths were improved by a factor of 20. The design has been developed to produce a versatile, general-purpose instrument for different excitation sources. Diffraction peaks can be measured at large angle without interference of the large optical elements close to the sample. The system described here has been commissioned and is now open to the general user community at the LCLS.
